# The impact of CFS/ME on employment and productivity in the UK: a cross-sectional study based on the CFS/ME national outcomes database

**DOI:** 10.1186/1472-6963-11-217

**Published:** 2011-09-15

**Authors:** Simon M Collin, Esther Crawley, Margaret T May, Jonathan AC Sterne, William Hollingworth

**Affiliations:** 1 School of Social & Community Medicine, Centre for Child & Adolescent Health, University of Bristol, Oakfield House, Oakfield Grove, BS8 2BN, UK; 2 School of Social & Community Medicine, Canynge Hall, 39 Whatley Road, BS8 2PS, UK

## Abstract

**Background:**

Few studies have investigated factors associated with discontinuation of employment in patients with CFS/ME or quantified its impact on productivity.

**Methods:**

We used patient-level data from five NHS CFS/ME services during the period 01/04/2006-31/03/2010 collated in the UK CFS/ME National Outcomes Database. We used logistic regression to identify factors associated with discontinuation of employment. We estimated UK-wide productivity costs using patient-level data on duration of illness before assessment by a CFS/ME service, duration of unemployment, age, sex and numbers of patients, in conjunction with Office for National Statistics income and population data.

**Results:**

Data were available for 2,170 patients, of whom 1,669 (76.9%) were women. Current employment status was recorded for 1,991 patients (91.8%), of whom 811 patients (40.7%) were currently employed and 998 (50.1%) had discontinued their employment "because of fatigue-related symptoms". Older age, male sex, disability, fatigue, pain, and duration of illness were associated with cessation of employment. In a multivariable model, age, male sex, and disability remained as independent predictors. Total productivity costs among the 2,170 patients due to discontinuation of employment in the years preceding assessment by a specialist CFS/ME service (median duration of illness = 36 months) were £49.2 million. Our sample was equivalent to 4,424 UK adults accessing specialist services each year, representing productivity costs to the UK economy of £102.2 million. Sensitivity analyses suggested a range between £75.5-£128.9 million.

**Conclusions:**

CFS/ME incurs huge productivity costs amongst the small fraction of adults with CFS/ME who access specialist services.

## Background

Chronic Fatigue Syndrome or Myalgic Encephalopathy (CFS/ME) is defined as persistent or recurrent debilitating fatigue that is not lifelong, the result of ongoing exertion, alleviated by rest, or explained by other conditions, and that results in a substantial reduction in activity [1-3]. CFS/ME is heterogeneous [4] and relatively common with an estimated prevalence from population surveys of between 0.2% and 2.6% [5-10]. Adults with CFS/ME are unwell for a long time, with a median duration of illness of 6.3 years [11].

Few studies have investigated the impact of CFS/ME on employment and productivity. A systematic review of the literature that collated employment data from a variety of studies between 1988 and 2001 found that 54% of patients with CFS/ME were unemployed [12]. Estimates of productivity costs to the US economy have varied widely, depending mainly on the estimated prevalence of CFS/ME in the communities surveyed: $9 billion (Kansas, 1994) [13], $17 billion (Illinois, 1995-1998) [14], $37 billion (Georgia, 2004-2005) [15]. No comparable analysis has been performed on data from the UK.

In the UK, specialist CFS/ME clinical teams provide a range of treatments according to National Institute for Health and Clinical Excellence (NICE) guidelines [3]. Access to these services throughout the UK is variable [16,17], and the proportion of people with CFS/ME who are referred to specialist services is unknown. Amongst those who are referred to a specialist service, the impact of their illness on employment and income has not been quantified. Here we use data on patients attending 5 specialist CFS/ME services in the UK to investigate the clinical and demographic factors associated with discontinuation of employment, to estimate earnings lost as a consequence of CFS/ME, and to estimate productivity costs to the UK economy incurred by patients with CFS/ME prior to assessment by a specialist service.

## Methods

### Study population

The UK CFS/ME National Outcomes Database (NOD) collects assessment and follow-up data on patients attending or visited by NHS specialist CFS/ME services. The aim of the NOD is to enable benchmarking and evaluation of these services. All contributing clinical teams are members of the British Association for CFS/ME (BACME). BACME comprises more than 40 adult and 12 paediatric teams, together assessing more than 6,000 adults and children each year. We included in our study all patients ≥ 18 years and ≤ 64 years old who had attended specialist CFS/ME services provided by clinical teams in Bristol (Frenchay), Wells (Somerset), Leeds (Leeds & West Yorkshire), Barts and The London, and Epsom and St Helier (South West London and Surrey) during the period 01/04/2006 to 31/03/2010. These teams had assessed and treated a constant number of patients each year during the study period. For each team, patients were referred with a diagnosis of CFS/ME and this was confirmed at assessment according to CDC diagnostic criteria [1,2]. Other teams that contributed data to the NOD during this period were excluded either because they contributed data for a small number of patients or because their CFS/ME service had been established relatively recently.

### Patient-level data

We collected the following data at the time of assessment by the CFS/ME service: age, sex, ethnicity, diagnostic criteria, and self-reported duration of illness (time elapsed, in months, between onset of symptoms and clinical assessment). Current employment status was recorded in 4 categories: i) "Currently employed full- or part-time"; ii) "Temporarily discontinued because of fatigue-related symptoms"; iii) "Indefinitely discontinued because of fatigue-related symptoms"; iv) "Other". In addition, patients completed the following inventories prior to assessment: 11-item Chalder Fatigue Scale [18]; 10-item SF-36 physical function subscale [19], 14-item Hospital Anxiety and Depression Scale (HADS) [20] and a Visual Analogue Pain Rating Scale (score of 0 for "no pain" and 100 for "pain as bad as possible"). The Chalder Fatigue Scale was scored using the 0-3 method for scoring each question (0 "Less than usual", 1 "No more than usual", 2 "More than usual", 3 "Much more than usual"). On the physical function sub-scale of the SF-36, patients scored 0 ("Yes, limited a lot") 5 ("Yes, limited a little") or 10 ("No, not limited at all") for each question, so that the most disabled patients scored 0 while those with unaffected physical function scored 100. Inventory total scores (and each HADS sub-scale score) were coded as missing if > 1 question was unanswered; if only one item was missing, an adjusted total score was calculated.

### Factors associated with discontinuation of employment

We used Chi-squared tests and Student's t-test to compare the characteristics (age, sex, duration of illness, fatigue, disability, anxiety, depression and pain) of patients who were in employment at time of assessment with the characteristics of patients who had temporarily or permanently discontinued employment (before assessment) due to their illness. These characteristics were included in a logistic regression model to identify factors independently associated with discontinuation of employment among patients for whom complete data on all characteristics were available. So that regression coefficients for the different inventories were comparable, they were rescaled so that the range for each was approximately 0 - 10.

### Discontinuation of employment

In May 2010, the NOD forms and questionnaires were amended to include more detailed questions about the effect of CFS/ME on employment, specifically, patients were asked to give the "Date when reduced hours/sick leave/unemployment began". We used responses to this question to estimate the duration of unemployment as a fraction of the duration of illness in an external sample of patients ≥ 18 years old assessed by any team contributing data to the NOD during the period 01/06/2010 to 30/11/2010. We used the mean value of this fraction in our calculations, stratified by sex but not by age (due to insufficient numbers in some of the age strata).

### Per patient productivity costs due to discontinuation of employment

Productivity costs were estimated using average annual earnings data by sex and age group obtained from the Office for National Statistics [21]. These were used to estimate age- and sex-specific cumulative loss of earnings due to discontinuation of employment prior to assessment by a NHS CFS/ME specialist clinical team. The total productivity costs in the patient sample were estimated as the product of: the number of patients assessed who had a new or confirmed diagnosis of CFS/ME; the fraction who had discontinued their employment; the median duration of illness; the proportional duration of unemployment (mean 'duration of unemployment'/mean 'duration of illness') from the external sample; and mean annual income. This product was divided by the number of patients to yield an estimated total loss per person.

### Nationwide productivity costs

Access to CFS/ME specialist assessment throughout the UK is variable [16,17]. The five CFS/ME teams contributing data to this study represent areas of the country that currently have higher levels of access. We used data from these five centres to extrapolate and estimate the potential nationwide productivity costs due to CFS/ME in patients prior to specialist assessment. The clinical teams in our study were asked to identify the Primary Care Trusts (PCTs) from which the majority of patients were referred. The annual proportion of the population who access specialist assessment was estimated by dividing the number of patients in the sample by the number of study years (four) and then by the estimated population in the PCTs served by the teams in our study in each age and sex stratum [22]. These proportions were multiplied by UK population estimates [22] to estimate the total population of people with CFS/ME in the UK who might receive specialist assessment each year. The nationwide productivity costs due to CFS/ME prior to specialist assessment were estimated for this population using age and sex stratified earnings data, as previously described. In two one-way sensitivity analyses, we used the lower and upper bounds of the 95% confidence intervals for the fractions of those who had discontinued their employment and the fraction 'duration of unemployment'/'duration of illness' to estimate upper and lower bounds for the total UK loss.

### Ethical approval

The North Somerset & South Bristol Research Ethics Committee decided that the collection and analysis of CFS/ME patient data were part of service evaluation and as such did not require ethical review by a NHS Research Ethics Committee or approval by NHS Research and Development offices (REC reference number 07/Q2006/48).

## Results

### Description of adult CFS/ME cohort in the National Outcomes Database

Clinical assessment data were available for 2,170 patients, of whom 1,669 (76.9%) were women. The mean ages of men and women were 41.4 years and 38.6 years, respectively (Student's t test P < 0.001). Current employment status was recorded for 1,991 patients (91.8%), of whom 811 patients (40.7%) reported their status as "Currently employed"; 322 (16.2%) as "Temporarily discontinued because of fatigue-related symptoms"; 676 (34.0%) as "Permanently discontinued because of fatigue-related symptoms"; and 182 (9.1%) as "Other". Of the 811 patients who were currently employed and the 998 patients (50.1%) who had discontinued their employment, complete questionnaire data (duration of illness, Chalder Fatigue, SF-36, HADS, and Visual Analogue Pain) were collected at time of clinical assessment for 641 (79.0%) and 756 (75.8%) patients, respectively.

### Factors associated with discontinuation of employment

Men, people in older age groups, and people who had been ill for longer were more likely to have ceased employment due to their fatigue-related symptoms (Table 1): the proportion of patients in the 18 - 21 year old age group who had discontinued their employment was 42.7%, compared with 65.4% of patients 50 - 59 years old; 60.3% of men were no longer employed, compared with 53.6% of women; median duration of illness was 4 years among patients who had ceased working, compared with 3 years among patients who were currently employed. There was an overall downward trend (P_trend _= 0.03) in median duration of illness among all patients between 2006 (42 months) and 2010 (36 months).

**Table 1:** Characteristics of CFS/ME patients in current and discontinued employment due to fatigue-related symptoms (IQR = interquartile range)

	Currently employed	Employment discontinued	P-value*
Age (years)	n = 811	n = 998	
18 - 21	59 (57.3%)	44 (42.7%)	
22 - 29	153 (49.8%)	154 (50.2%)	
30 - 39	265 (51.5%)	250 (48.5%)	
40 - 49	202 (42.9%)	269 (57.1%)	
50 - 59	119 (34.6%)	225 (65.4%)	
60+	13 (18.8%)	56 (81.2%)	< 0.001
Sex	n = 811	n = 998	
Female	643 (46.4%)	743 (53.6%)	
Male	168 (39.7%)	255 (60.3%)	0.02
Duration of illness (months)	n = 743	n = 878	
Median (IQR)	35 (15 - 84)	48 (18 - 120)	< 0.001
Chalder Fatigue (0 - 33)	n = 752	n = 911	
Median (IQR)	25 (21 - 30)	27 (23 - 31)	< 0.001
SF-36 physical function (scale 0 - 100)	n = 744	n = 901	
Median (IQR)	50 (35 - 65)	30 (20 - 45)	< 0.001
HADS anxiety (scale 0 - 21)	n = 751	n = 916	
Median (IQR)	10 (7 - 13)	11 (7 - 14)	0.02
HADS depression (scale 0 - 21)	n = 753	n = 919	
Median (IQR)	9 (6 - 12)	10 (7 - 13)	< 0.001
Visual Analogue Pain (scale 0 - 100)	n = 717	n = 886	
Median (IQR)	43 (18 - 65)	55 (26 - 72)	< 0.001

* Chi-squared test for categorical variables; Mann-Whitney two-sample test for continuous variables

The clinical characteristics (fatigue, disability, anxiety, depression, and pain) of those who had ceased working indicated a greater severity of illness than was experienced by people who were currently employed. Most notably, disability (as measured by the SF-36 physical function questionnaire) showed a 20-point difference (30 compared with 50) on a scale of 0 - 100, and pain (measured on a 0 - 100 visual analogue pain rating scale) showed a 12-point difference (55 compared with 43).

In logistic regression models (adjusted for centre and year of assessment): older age, longer duration of illness, worse fatigue, poorer physical function, and pain were strongly associated with cessation of employment; male sex, greater anxiety and depression were weakly associated with cessation of employment (Table 2). In a multivariable logistic regression model that included all of these variables (Table 2), older adults and men were more likely to have discontinued their employment, but the only clinical characteristic associated with unemployment was poor physical function. Mutual adjustment for all patient characteristics slightly attenuated the association of older age with discontinuation of employment, but the association with sex was strengthened. The association of SF-36 score with discontinuation of employment was unaffected in strength and magnitude.

**Table 2:** Associations of CFS/ME patient characteristics with discontinuation of employment: analyses based on patients with complete data for all variables in table (N = 1,397 of whom 641 (45.9%) currently employed, 756 (54.1%) discontinued employment)

Variable*	Partially-adjusted odds ratio** (95% CI)	P-value	Fully-adjusted odds ratio*** (95% CI)	P-value
10-year age group	1.31 (1.19, 1.44)	< 0.001	1.16 (1.05, 1.29)	0.005
Sex (female *vs *male)	0.76 (0.59, 0.98)	0.03	0.62 (0.46, 0.82)	0.001
Duration of illness (years)	1.03 (1.01, 1.05)	0.003	1.01 (0.99, 1.03)	0.36
Chalder Fatigue	1.20 (1.12, 1.29)	< 0.001	1.02 (0.93, 1.11)	0.68
SF-36 (physical function)	0.70 (0.66, 0.74)	< 0.001	0.71 (0.67, 0.76)	< 0.001
HADS anxiety	1.04 (1.00, 1.09)	0.06	0.98 (0.92, 1.04)	0.42
HADS depression	1.18 (0.98, 1.25)	0.06	1.05 (0.98, 1.14)	0.16
Visual Analogue Pain	1.12 (1.08, 1.17)	< 0.001	1.00 (0.96, 1.05)	0.84

* Chalder Fatigue, SF-36, HADS, and Visual Analogue Pain measures were re-scaled to range from 0 to 10. For SF-36, odds ratio < 1 indicates that higher levels of disability are positively associated with discontinuation of employment.

** Adjusted only for centre and year of assessment (for continuous measures, odds ratio is per unit increase)

*** Adjusted for centre, year of assessment and all variables in table (for continuous measures, odds ratio is per unit increase)

### Discontinuation of employment

Date of discontinuation of employment was available for 60 patients (22 male, 38 female) who were assessed between 01/06/2010 and 30/11/2010 for whom data had been obtained from the amended assessment forms. Median (interquartile range (IQR)) of duration of illness was 26 (14 - 108) months; median (IQR) of 'duration of unemployment' was 11 (5 - 26) months. The fraction 'duration of unemployment'/'duration of illness' had a mean value of 0.52 (95% CI 0.38, 0.66) for men and 0.47 (95% CI 0.35, 0.59) for women, indicating that discontinuation of employment occurred, on average, at the midpoint of the interval between onset of illness and assessment by a specialist CFS/ME service for both sexes.

### Productivity costs due to discontinuation of employment

Data on employment status and duration of illness were available for 82.4% (413/501) and 82.5% (1,377/1,669) of men and women, respectively. Patients missing these data were slightly older (mean age 41.0 years *vs *38.8 years, t test P = 0.002) and had slightly higher Chalder Fatigue scores (26.3 *vs *24.4, t test P < 0.001) than those with complete data, but were similar in all other characteristics. The estimated productivity costs due to discontinuation of employment based on data collected from 2,170 patients (501 men and 1,669 women) attending the five CFS/ME specialist services in our study between 04/2006 and 03/2010 are shown in Table 3.

**Table 3:** Estimated loss of productivity due to CFS/ME based on patients referred to 5 NHS specialist services (2006 - 2010)

Sex and age (years)	N^a^	Discontinued employment (%)^b^	Median duration of illness (months)^b^	Mean annual income (£)^c^	Total loss of productivity (£)^d^	Loss per person (£)^e^	Population in PCTs in study^f^	Proportion of population who access services (per 100,000 per year)	UK population (1000's)	Number of people who might access services per year (UK)	Productivity costs among people who might access services (UK)
											
Male											
18 - 21	26	40%	21	11,546	109,271						
22 - 29	72	48%	30	23,460	1,054,011	11,870	655,664	3.74	5,135	192	2,277,502
30 - 39	128	53%	72	35,172	7,444,534	58,160	554,200	5.77	4,069	235	13,663,983
40 - 49	127	56%	60	39,390	7,283,684	57,352	535,400	5.93	4,523	268	15,383,619
50 - 59	116	65%	48	36,590	5,738,483	49,470	414,100	7.00	3,690	258	12,782,017
60 - 64	32	59%	31	26,505	672,223	21,007	190,800	4.19	1,818	76	1,601,287
Subtotals	501				22,302,206	44,515	2,350,164	5.33	19,234	1,030	45,708,408
											
Female											
18 - 21	150	25%	36	8,875	469,266						
22 - 29	294	43%	26	18,508	2,382,677	6,423	638,346	17.39	4,922	856	5,497,849
30 - 39	461	43%	36	22,047	6,162,231	13,367	548,400	21.02	4,086	858	11,475,526
40 - 49	411	53%	48	20,880	8,550,786	20,805	541,600	18.97	4,631	879	18,280,147
50 - 59	281	61%	55	19,893	7,345,405	26,140	423,200	16.60	3,796	630	16,469,450
60 - 64	72	60%	87	13,662	2,011,101	27,932	200,000	9.00	1,901	171	4,779,382
Subtotals	1,669				26,921,466	16,130	2,351,546	17.74	19,335	3,394	56,502,353
											
Totals	2,170				49,223,672	22,684	4,701,710	11.54		4,424	102,210,761

a) Numbers of patients with CFS/ME assessed by 5 specialist services that had contributed constant patient numbers each year to the CFS/ME National Outcomes Database over the period 04/2006 - 03/2010

b) Based on subset of patients (413 male, 1,377 female) for whom data on occupation and duration of illness were recorded in the CFS/ME National Outcomes Database

c) Source: Office for National Statistics "Annual Survey of Hours and Earnings, 2009"

d) (N × fraction unemployed × (median duration of illness × F) × (mean annual income/12)) where F = 0.52 for men, F = 0.47 for women (F = duration of unemployment/duration of illness)

e) The 18-21 and 22-29 age groups were combined into a single 18-29 age group to match PCT population age strata

f) Source: Office for National Statistics "Mid-2008 Population Estimates by Primary Care Organization"

The total productivity costs among the 2,170 patients in our study were £22.3 million for men (equivalent to £44,515 per patient) and £26.9 million for women (equivalent to £16,130 per patient), giving a total loss of £49.2 million (equivalent to £22,684 per patient). The prevalence of CFS/ME referred for specialist assessment was much higher in women (17.7 per 100,000) than in men (5.3 per 100,000) in the five CFS/ME services in our study; the overall prevalence of CFS/ME referred for specialist assessment was 11.54 per 100,000. By extrapolating these estimates to the UK population, we estimated that each year 4,424 working age adults with CFS/ME might be referred for specialist assessment, and that this group would already have incurred productivity costs of £102.2 million due to their illness by the time of the assessment.

The lower and upper bounds of the 95% confidence intervals for the fraction 'duration of unemployment'/'duration of illness' (0.38 - 0.66) in men, (0.35 - 0.59) in women, corresponded to productivity costs of £75.5 - £128.9 million per year; the lower and upper bounds of the 95% confidence intervals for the proportions unemployed corresponded to productivity costs of £86.2 - £117.5 million per year.

## Discussion

This is the first study to estimate the productivity costs of patients with CFS/ME who access specialist services. We have estimated an annual productivity cost to the UK economy of approximately £100 million. The total productivity costs of CFS/ME are likely to be substantially higher because many adults with CFS/ME are not referred to specialist clinical teams. Our estimates for the proportion of the population who access specialist services are 20-40 times lower than the estimated prevalence of CFS/ME among adults in the USA based on community surveys [8,23]. Although we found a decrease in duration of illness over the study period, suggesting that awareness and availability of CFS/ME specialist services improved during the period of our study, the median duration of illness in 2010 (36 months) was still much longer than the 6 months recommended by NICE for referral of mild cases of CFS/ME to specialist services [3]. The situation in those parts of the UK that are not covered by established specialist CFS/ME services is likely to be worse.

The strength of our study is that it is based on routinely-collected clinical data from several teams across the UK. Hence, the patients in our study are likely to be representative of adults who have been referred to CFS/ME specialist services within the NHS over the past 4 years. We had no data with which to assess the rate at which people with CFS/ME recover and return to work, either with or without specialized treatment. According to a systematic review of the literature, the proportion of adults in employment increased following interventions for CFS/ME (individualised rehabilitation, cognitive behavioural therapy and exercise therapy) and decreased in observational studies with no intervention [12]. Evidence from a recent evidence trial of cognitive behavioural therapy and graded exercise therapy indicated a recovery rate of 30-40% one year after treatment [24]. Our analysis was based on income data from 2009, which we applied retrospectively to patients who may have discontinued their employment several years earlier, e.g. female patients in the 60 - 64 years age group assessed in 2006 with a median duration of illness of 7 years were assumed to have discontinued employment in 2002. We do not know whether socioeconomic status is associated with CFS/ME and with access to services, therefore we were unable to weight our loss of earnings estimates. Also, by valuing time off work using average earnings we are assuming that the worker is not replaced, for example from those currently unemployed, and that there is a long-term cost to society. Alternative friction-cost approaches to valuing lost productivity would result in a lower estimate of the productivity costs of CFS/ME [25].

The proportion of patients in our study who had discontinued employment (50%) is consistent with an estimate from a systematic review of unemployment due to CFS (54%) [12]. That disability was the main independent predictor of discontinuation of employment in a multivariable model is consistent with a study of employees on long-term sick leave due to fatigue [26]. Our finding suggests that people with CFS/ME continue in employment despite the primary (fatigue and pain) and secondary effects (depression and anxiety) of CFS/ME. Instead, loss of physical capacity is the ultimate arbiter of inability to continue working. That women and younger people are less likely to discontinue work is consistent with findings from a US community-based study [13]. A UK-based study of patients presenting with chronic fatigue in a primary care setting reported that loss of employment over the preceding 6-month period had a mean cost per person of £2,350 (95% CI £1734 - £2966) although it is unclear whether all of these patients had CFS/ME [27]. The equivalent cost from our data (based on the weighted average of half the mean annual income) would be £5,753. This figure may indicate the higher productivity costs of CFS/ME *vs *chronic fatigue [28]. The same study estimated that productivity costs represent 61% of the total costs of CFS/ME; the remaining costs were attributed to health resource use (13%) and informal care (26%) [27].

## Conclusion

The main implication of our findings is that effects on employment and productivity must be accounted for in estimates of the cost-effectiveness of CFS/ME interventions and service provision. Screening for CFS/ME by general practitioners and occupational therapists could be cost-effective in conjunction with early intervention for treating CFS/ME. We have quantified the impact of CFS/ME among patients who access specialist CFS/ME services in terms of loss of employment and productivity, i.e. as an indirect cost to the UK economy. In addition to this indirect cost, health resource use and welfare payments impose direct costs, and families of patients must bear the costs of informal care, often reducing their own working hours [27]. In young adults, disruption of education reduces productivity in later years [15]. Above and beyond these financial costs, CFS/ME has a huge impact on quality of life [29]. Research is urgently needed to establish whether wider access and earlier referral to specialist CFS/ME services is a cost-effective way of reducing productivity losses.

## Competing interests

EC is medical advisor for Association of Young People with ME (AYME).

## Authors' contributions

EC had the original idea for the study; SC performed all statistical analyses with guidance and support from MM, JS and WH; SC and EC wrote the first draft and incorporated comments from all authors; all authors contributed to the interpretation and writing of the paper, and read and approved the final manuscript.

## Pre-publication history

The pre-publication history for this paper can be accessed here:

http://www.biomedcentral.com/1472-6963/11/217/prepub
