# Correction: The impact of CFS/ME on employment and productivity in the UK: a cross-sectional study based on the CFS/ME national outcomes database

**DOI:** 10.1186/s12913-023-09484-7

**Published:** 2023-05-10

**Authors:** Simon M Collin, Esther Crawley, Margaret T May, Jonathan AC Sterne, William Hollingworth, for UK CFS/ME National Outcomes Database

**Affiliations:** 1 School of Social & Community Medicine, Centre for Child & Adolescent Health, University of Bristol, Oakfield House, Oakfield Grove, Bristol, BS8 2BN UK; 2 School of Social & Community Medicine, University of Bristol, Canynge Hall, 39 Whatley Road, Bristol, BS8 2PS UK

**Correction:** **BMC Health Services Research 11, 217 (2011)**

**https://doi.org/10.1186/1472-6963-11-217**

Following a report of a publications review jointly commissioned by the Health Research Authority and the University of Bristol, the authors would like to correct the following element of the ethics statement provided in the original article [1]:

The data used in this study were originally collected and collated in the National Outcomes Database for the purposes of evaluation of CFS/ME services. The collection of a subset of CFS/ME patient data as part of the national CFS/ME collaborative was confirmed to be service evaluation by the North Somerset & Bristol Research Ethics Committee under REC reference 07/Q2006/48, and in a letter dated 29 January 2007 the Chair of the Research Ethics Committee had previously confirmed (a) that it would not be necessary to apply for ethical permission to use the data being collected as part of service evaluation for the national CFS/ME collaborative and (b) that if in future these data were to be used as part of a research project, this would be agreeable.

The original article [1] has been corrected.
